# Electrocatalytic oxidation of Epinephrine and Norepinephrine at metal oxide doped phthalocyanine/MWCNT composite sensor

**DOI:** 10.1038/srep26938

**Published:** 2016-06-01

**Authors:** Ntsoaki G. Mphuthi, Abolanle S. Adekunle, Eno E. Ebenso

**Affiliations:** 1 Material Science Innovation & Modelling (MaSIM) Research Focus Area, Faculty of Agriculture, Science and Technology, North-West University (Mafikeng Campus), Private Bag X2046, Mmabatho 2735, South Africa; 2 Department of Chemistry, Obafemi Awolowo University, Ile-Ife, Nigeria

## Abstract

Glassy carbon electrode (GCE) was modified with metal oxides (MO = Fe_3_O_4_, ZnO) nanoparticles doped phthalocyanine (Pc) and functionalized MWCNTs, and the electrocatalytic properties were studied. Successful synthesis of the metal oxide nanoparticles and the MO/Pc/MWCNT composite were confirmed using FTIR, Raman and SEM techniques. The electrodes were characterized using cyclic voltammetry (CV) technique. The electrocatalytic behaviour of the electrode towards epinephrine (EP) and norepinephrine (NE) oxidation was investigated using CV and DPV. Result showed that GCE-MWCNT/Fe_3_O_4_/2,3-Nc, GCE-MWCNT/Fe_3_O_4_29H,31H-Pc, GCE-MWCNT/ZnO/2,3-Nc and GCE-MWCNT/ZnO/29H,31H-Pc electrodes gave enhanced EP and NE current response. Stability study indicated that the four GCE-MWCNT/MO/Pc modified electrodes were stable against electrode fouling effect with the percentage NE current drop of 5.56–5.88% after 20 scans. GCE-MWCNT/Fe_3_O_4_/29H,31H-Pc gave the lowest limit of detection (4.6 μM) towards EP while MWCNT/ZnO/29H,31H-Pc gave the lowest limit of detection (1.7 μM) towards NE. The limit of detection and sensitivity of the electrodes compared well with literature. Electrocatalytic oxidation of EP and NE on GCE-MWCNT/MO/Pc electrodes was diffusion controlled with some adsorption of electro-oxidation reaction intermediates products. The electrodes were found to be electrochemically stable, reusable and can be used for the analysis of EP and NE in real life samples.

Norepinephrine (NE) is an important catecholamine neurotransmitter in the mammalian central nervous system. It is secreted and released by the adrenal glands, the noradrenergic neurons during synaptic transmission and relinquished as a metabotropic neurotransmitter from nerve endings in the sympathetic nervous system and some areas of the cerebral cortex^1^,^2^. The human adrenal medulla releases about 20% NE, while the adrenergic neurons are responsible for the major NE production^3^. NE is responsible for increased heart rate, blood pressure, dilation of pupil, and dilation of air passage in lungs and narrowing of blood vessels. NE is important for attention and focus, learning, memory, and the sleep–wake cycle; hence it is also used as performance enhancing drug in competitive games by athletes; therefore prohibited by the World Anti-Doping Agency^4^,^5^. It promotes the conversion of glycogen to glucose in the liver and helps in converting the fats into fatty acids, resulting in an increment in energy production^4^. Extreme abnormalities of NE concentration levels caused by its metabolic dysfunction may lead to occurrence of many pathological conditions such as thyroid hormone deficiency, congestive heart failure, arrhythmias and idiopathic postural hypotension^6^,^7^.

Epinephrine (EP) also known as adrenaline exists as an organic cation^8^, which is about nmolL^−1^ in human serum^9^. It belongs to the family of inhibitory/catecholamine neurotransmitters in mammalian central nervous systems. It was first discovered by Takamine and Aldrich in 1901, and synthesized by Stolz and Dalkin in 1904^10^. EP acts as a cellular chemical messenger^11^, and many diseases are related to the change of its concentration. It is a hormone synthesized by the adrenal medulla of the adrenal glands^12^,^13^,^14^, it stimulates a series of actions of the sympathetic nervous system called “fight or flight” response^15^. EP controls the performance of the nervous system, and its abnormal levels affect the regulation of the blood pressure heart rate, and glycogen metabolism^8^,^16^. Determination of the level of EP is important for diagnosis of Parkinson’s disease, among other mental disorders^17^. It has been used as a common healthcare medicine, for instance EP drugs are used to treat anaphylactic shock, bronchial asthma and organic heart disease^18^. Therefore, a quantitative determination of EP at physiological pH in our body fluids has become increasingly significant.

Carbon nanotubes (CNTs) are an important class of materials due to their unique electronic, mechanical, and structural characteristics. The physical and catalytic properties make CNTs ideal for use as chemical sensors and for electrochemical detection^18^. However their electrochemical properties are sensitive when chemically doped with various molecules^19^. It has also been reported that the application of pristine CNTs is limited due to their poor solubility in both organic and inorganic solvents^20^ and also the CNTs walls are not reactive^19^; therefore it is of best interest to attach functional groups such as –COOH, −OH or −C = O to their surface to optimize their use and increase their solubility^21^. The mechanically strong, small size and chemically stable nature of CNTs is very appealing for sensing applications^22^. Furthermore, CNT based platforms were cited as biocompatible sensors because of the similarity in size with analytes such as cells, proteins and even DNA; they have shown a significant development because of their promising applications in nanoelectronics as well as in highly sensitive biosensors^18^. The first electrochemical application of CNTs was carried out by Britto, where multi-walled carbon nanotubes (MWCNTs) were used to study the electrochemical oxidation of DA^23^. The results obtained from the study showed a two electron transfer redox reversible process and the oxidation of DA showed a low potential with a faster rate than that observed for graphite electrodes. These remarkable results suggest that MWCNTs possess properties such as high electrical conductivity, larger surface-active groups to-volume ratio, chemical stability and significant mechanical strength, as a consequence, MWCNTs can serve as excellent substrates for the development of biosensor devices^24^. From 1996 the application CNTs in electrochemistry increased severely towards detection of biological analytes and gases using sensors or biosensors.

There is currently an interest in the use of metal oxide nanoparticles (NPs), metal-doped metal oxides, metal oxide-CNTs nanocomposites, and metal oxide-polymer composites^25^ in electrochemistry to improve the performance of electrochemical detection of biological and environmental analytes^1^. Analytical devices based on nanostructured metal oxides are cost-effective, highly sensitive due to the large surface-to-volume ratio of the nanostructure and additionally show excellent selectivity^25^. Metal and metal oxides NPs have been used to modify electrodes for use as electrocatalysts and biosensors; hence they play an important role in diagnostic devices^26^. Shan *et al*.^27^ investigated Fe_3_O_4_/chitosan modified electrode as a feasible sensor for detection of H_2_O_2_. He found that there was limited interference, prompt response, good reproducibility and the electrode showed long term stability, therefore he suggested that these might be beneficial for future environmental and biological applications towards detection of H_2_O_2_. In addition Adekunle *et al*.^28^ conducted a study on the voltammetric detection of DA using easily prepared nano-scaled iron oxide (Fe_2_O_3_) catalyst supported on MWCNTs modified pyrolytic graphite electrode.

Phthalocyanines (Pcs) and their derivatives are well-known blue-green organic semiconductor materials that belong to the family of aromatic heterocyclic conjugated molecules, with alternating single-double bond structures^29^. They are made of delocalised π-electrons system^30^, where π-electron delocalisation and interactions with central metal atom on metal phthalocyanines MPcs determine the redox properties^31^. Their thermal and chemical stability are important properties that make them molecules to be incorporated into electrochemical sensors. Furthermore the possibility of incorporating about 70 different metal atoms into their ring^32^, and also being able to have variation of the substituents in the side chain, can allow production of peculiar and optimized thin films having different degrees of sensitivity, selectivity and stability^33^. Wang^34^ reported that among the metal substituted Pc, lead-Pc is the most interesting one in terms of reproducibility, fast response recovery and excellent gas sensitivity. Pcs are capable of binding nonspecifically with various analytes through van der Waal forces, hydrogen bonding, and coordination interactions with the central metal^35^.

This study explored the unique electrocatalytic properties of GCE-MWCNT/MO/Pc modified electrodes towards the oxidation of epinephrine (EP) and Norepinephrine (NP). In this work, we have shown that the fabrication of GCE-MWCNT/Fe_3_O_4_/2,3-Nc, GCE-MWCNT/Fe_3_O_4_29H,31H-Pc, GCE-MWCNT/ZnO/2,3-Nc and GCE-MWCNT/ZnO/29H,31H-Pc modified electrodes is simple and successful. We also demonstrated that the nanocomposite modified electrodes enhanced EP and NP oxidation current compared with other electrode studied. This study showed that GCE-MWCNT/ZnO/29H,31H-Pc was the best electrodes towards EP and GCE-MWCNT/ZnO/29H,31H-Pc was the best towards NP in terms of limit of detection. The two electrodes have also provided well-defined voltammograms for simultaneous detection of EP or NP without interference from amino acid (AA).

## Experimental

### Materials and reagents

Glassy carbon electrode (3 mm diameter) was purchased from CH Instrument USA. Polishing pads were obtained from Buehler, IL, USA and Alumina micro powder (1.0, 0.3 and 0.05 μm alumina slurries) was used for polishing the glassy carbon electrode (GCE). Pristine multi-walled carbon nanotubes (95% purity, 10–20 nm); Iron(III) chloride (FeCl_3_), zinc nitrate hexahydrate (Zn(NO_3_)_2_.6H_2_O), 29H,31H-Phthalocyanine (Pc), 2,3-Naphthalocyanine (Nc), (±)-Epinephrine hydrochloride, L-Norepinephrine hydrochloride, dopamine hydrochloride, ascorbic acid and other reagents are of analytical grade and obtained from Sigma-Aldrich, Merck chemicals and LABCHEM respectively. Ultra-pure water of resistivity 18.2 MΩ was obtained from a Milli-Q Water System (Millipore Corp., Bedford, MA, USA) and was used throughout for the preparation of solutions. A phosphate buffer solution (PBS) of 7.0 was prepared with appropriate amounts of NaH_2_PO_4_.2H_2_O, Na_2_HPO_4_.2H_2_O, and H_3_PO_4_, and adjusted with 0.1 M H_3_PO_4_ or NaOH. Prepared solutions were purged with pure nitrogen to eliminate oxygen and prevent any form of external oxidation during every electrochemical experiment.

### Synthesis of zinc oxide (ZnO) nanoparticles

Sodium hydroxide (NaOH) was dissolved in deionised water to a concentration of 1.0 M and the resulting solution was heated, under constant stirring, to the temperature of 70 °C. After achieving this temperature, a solution of 0.5 M Zn(NO_3_)_2_.6H_2_O was added slowly (drop wise for 60 minutes) into the NaOH aqueous solution under continuous stirring. In this procedure the reaction temperature was constantly maintained at 70 °C. The suspension formed with the dropping of 0.5 M Zn(NO_3_)_2_ · 6H_2_O solution to the alkaline aqueous solution was kept stirred for two hours at 70 °C. The material formed was filtered and washed several times with deionised water. The washed sample was dried at 65 °C in the oven for 24 hours to obtain the dry powder^36^.

### Synthesis of iron oxide (Fe_3_O_4_) nanoparticles

A mixture of FeCl_3_ stock solutions (30 mL) 2.0 M, Na_2_SO_3_ stock solution (20 mL) 1.0 M and concentrated ammonia (50.8 mL), diluted to a total volume of 800 mL was used. Fe^3+^ and SO_3_^2−^ were mixed; the colour of the solution changed from light yellow to red, indicating formation of a complex ion. The solution was quickly poured into the diluted ammonia solution under vigorous stirring, and the colour changed from red to yellow again. A black precipitate was formed and stirring continued for 30 minutes. After the reaction, the beaker containing the suspension was placed on a permanent magnet. The supernatant liquid was decanted and fresh deionised water was added into the beaker. This procedure was repeated several times until most of the ions in the suspensions were removed. The dry powder was obtained by filtration and drying at room temperature^37^.

### Preparation of MWCNT/metal oxide/phthalocyanine hybrids

Fe_3_O_4_ nanoparticles (2.5 mg) was dissolved in 1 mL DMF along with 2.0 mg of either of phthalocyanine, 2,3-Nc (naphthalocyanine) or 29H,31H-Pc; the solution was allowed to mix with the aid of ultrasonication for 1 hr. 2.0 mg of MWCNTs was dispersed in the DMF solution containing 2.5 mg of Fe_3_O_4_ nanoparticles and 2.0 mg of phthalocyanine. After ultrasonication, the Fe_3_O_4_/phthalocyanine hybrids were allowed to adsorb onto MWCNTs by spontaneous adsorption and sonication for 5h to give MWCNT/Fe_3_O_4_/phthalocyanine hybrids^24^. MWCNT/ZnO/phthalocyanine hybrids were prepared using the same procedure.

### Equipment and procedure

Scanning electron microscopy (SEM) experiment was performed using Zeiss Ultra Plus 55 HRSEM (Germany). FTIR experiments were carried out using Fourier transformed infrared spectrophotometer (Agilent Technology, Cary 600 series FTIR spectrometer, USA), while the Raman spectra were obtained using Xplora Horiba Raman Spectrometer (Olympus BX41 microscope, UK).

Electrochemical experiments were carried out using an Autolab Potentiostat PGSTAT 302 (Eco Chemie, Utrecht, and The Netherlands) driven by the GPES software version 4.9. A Ag|AgCl in saturated KCl and platinum wire were used as reference and counter electrodes respectively. A bench top Crison pH meter, Basic 20+ model, was used for pH measurements. All experiments were performed at 25 ± 1 °C while the solutions were de-aerated before every electrochemical experiment.

### Electrode modification procedure

Electrode modification was carried out using the drop-dry method. The glassy carbon electrode was cleaned by gentle polishing in aqueous slurry of alumina nanopowder on a silicon carbide-emery paper followed by a mirror finish on a Buehler felt pad. The electrode was further sonicated in double distilled water, and then absolute ethanol for 2 min to remove residual alumina particles that were trapped on the surface and dried at room temperature. Separate suspensions of the individual metal oxide nanoparticles, phthalocyanines and MWCNT were prepared by dissolving 5 mg of each in 1 mL of DMF and sonicated for 1 hr. Similarly, a composite of the catalyst was prepared by mixing 5 mg each of MWCNT with 5 mg either of the metal oxide (MO) nanoparticles (ZnO or Fe_3_O_4_) in 1 mL DMF, or by mixing 5 mg each of the three materials (phthalocyanines, MWCNT and MO) in 1 mL DMF^24^,^38^. 10 μL drops of the prepared suspensions were dropped on the bare GCE and dried in an oven at 50 °C for 5 min to obtain GCE-MWCNT, GCE-ZnO, GCE-Fe_3_O_4_, GCE-Pc electrodes. Other electrodes prepared are GCE-MWCNT/Fe_3_O_4_/2,3-Nc, GCE-MWCNT/Fe_3_O_4_/29H,31H-Pc, GCE-MWCNT/ZnO/2,3-Nc and GCE-MWCNT/ZnO/29H,31H-Pc. The electro active species surface coverage area of the electrodes was determined in 5 mM [Fe(CN)_6_]^3−/4−^ redox probe and estimated using the relationship, Γ = Q/nFA^39^.

## Results and Discussion

### Microscopic and spectroscopic results

#### FTIR and Raman spectroscopy results

**Figure 1:**
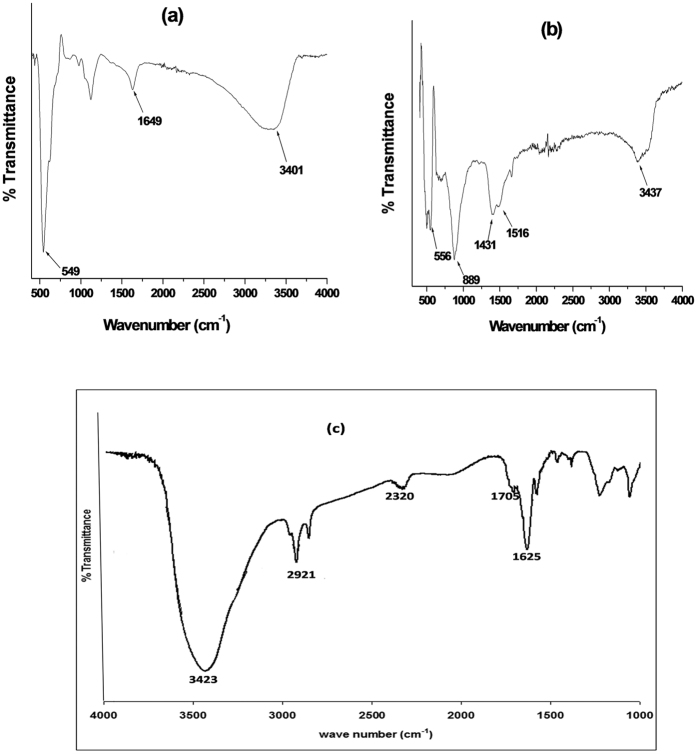
FTIR spectra of (**a**) Fe_3_O_4_, (**b**) ZnO and (**c**) MWCNT-COOH.

Figure 1 show the FTIR spectra of synthesized nanoparticles and functionalized MWCNT. The FTIR spectra of Fe_3_O_4_ nanoparticles (Fig. 1a) showed absorption peak at 549 cm ^−1^ which is attributed to the Fe-O stretching vibrations. The peak observed at 1649 cm^−1^ is related to the vibration of adsorbed water molecules, the H-OH group^40^ and the absorption peak at 3401 cm^−1^ is associated with of OH stretching vibration which also suggests the presence of some ferric hydroxide in Fe_3_O_4_^41^. Figure 1b shows the FTIR spectra of ZnO NPs, with Zn-O absorption band at 556 cm^−1^, and the peak at 3437 cm^−1^ indicating the presence of –OH stretching due to atmospheric moisture^42^. Other observed absorption peaks might be attributed to intramolecular hydrogen bonds^43^. The FTIR spectrum of functionalized MWCNT which identifies the functional groups attached to the surface and the sidewalls of MWCNTs is shown in Fig. 1c. The absorption peak observed at 1625 cm^−1^ correlates with stretching vibration of the C = C or C–O group attached to the surface of MWCNTs, and the absorption band at 1705 cm^−1^ is due to the C = O of the COOH groups. The peak at 2320 cm^−1^ arises from the graphitic component of MWCNTs^44^. The absorption band around 3423 cm^−1^ is associated with the O-H stretch of the terminal carboxylic group^45^.

**Figure 2:**
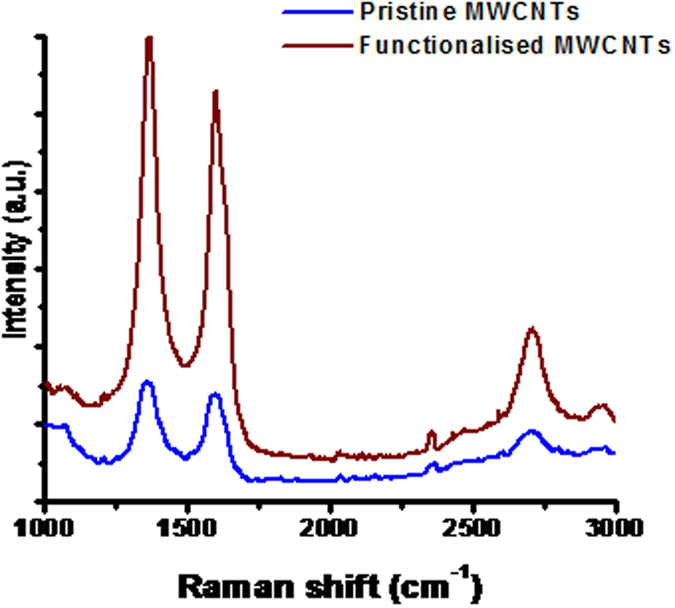
Raman spectra of pristine and functionalized MWCNTs.

Raman spectroscopy is a good technique used to study the low energy elementary excitation in materials. It is also used to characterize structural, electronic, vibrational and magnetic properties of carbon nanotubes^45^. The Raman spectra in Fig. 2 showed the D-band of functionalised and pristine MWCNTs at 1364 cm^−1^ and 1346 cm^−1^ respectively. This D-band is known to be associated with disorder-induced of CNTs^46^ which is due to the finite or nanosized graphitic planes and other forms of carbon^47^. The observed large intensity of the D-band peak as compared to the G-band intensity in CNTs indicates the presence of amorphous carbon^48^. Furthermore the D band in MWCNTs is attributed to defects in the tubes walls, vacancies, heptagon-pentagon pairs, kinks and heteroatoms^47^,^49^. The two G-bands observed appeared at 1598 cm^−1^ and 1587 cm^−1^ for functionalised and pristine MWCNTs respectively which represent E_2g_ tangential stretching mode of ordered crystalline graphitic and in-plane vibrations of sp^2^ hybridized carbon^45^,^46^. The D’-band on the shoulder of the G-band, which is not clearly visible on these spectra, occurred at 1631 cm^−1^ and 1628 cm^−1^ for functionalised and pristine MWCNTs respectively. This band is attributed to double resonance feature induced by disorder and defects^47^. The intensity ratio of the D-to-G band (I_D_/I_G_) was 0.85 and 0.8 for functionalised and pristine MWCNTs. The intensity ratio was calculated to evaluate the degree of disorder of walls of MWCNTs and the results obtained for the density ratio of functionalized is higher than that of pristine MWCNTs suggesting that functionalized MWCNTs walls had a higher degree of disorder. Theodore^45^ mentioned that an increase in I_D_/I_G_ indicates a higher degree of disorder, hence the G-band on functionalised MWCNTs is less affected by defects as compared to the D-band, therefore functionalised MWCNTs has functional groups attached to the surface and the walls, it is thus expected of functionalised MWCNTs to have a higher degree of disorder.

### SEM Characterization

**Figure 3:**
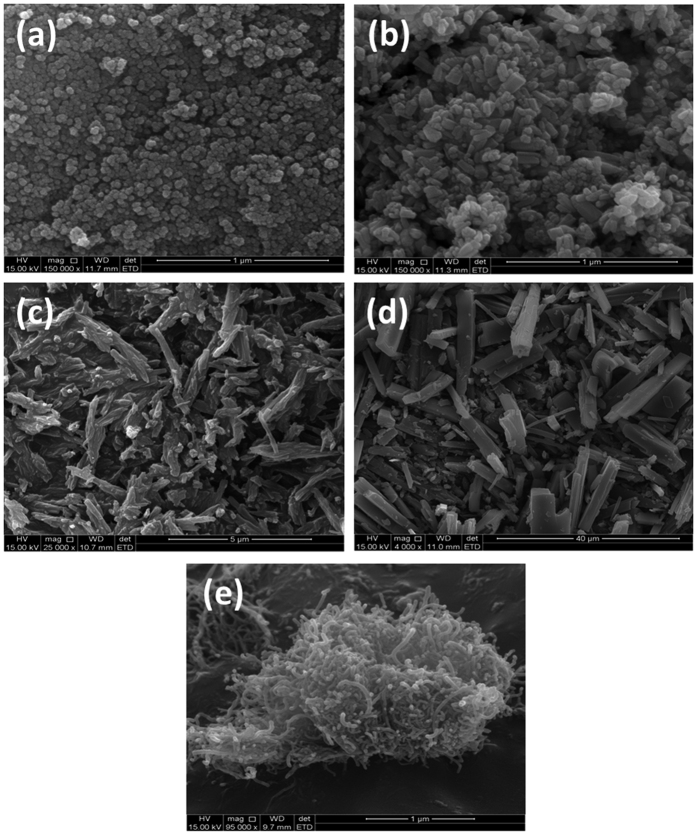
SEM images of (**a**) Fe_3_O_4_, (**b**) ZnO, (**c**) 2,3-Nc, (**d**) 29H,31H-Pc, (**e**) MWCNT.

**Figure 4:**
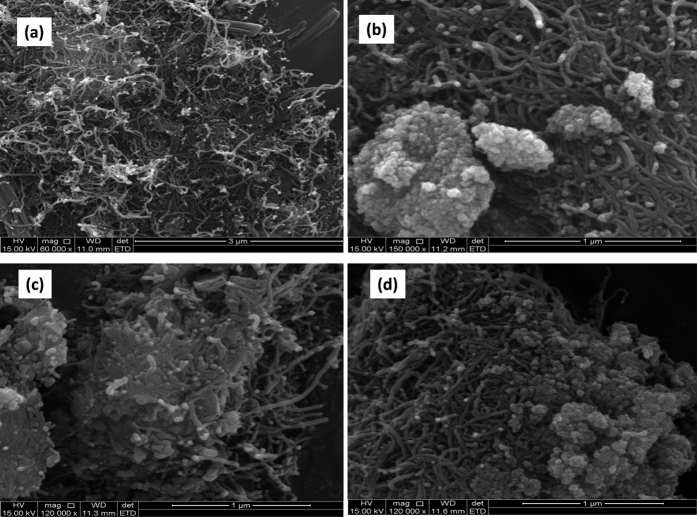
SEM images of (**a**) MWCNT/ZnO/2,3-Nc, (**b**) MWCNT/Fe_3_O_4_/2,3-Nc (**c**) MWCNT ZnO/29H,31H-Pc and (**d**) MWCNT/Fe_3_O_4_/29H,31H-Pc.

The morphology and microstructures of prepared compounds and their composites were characterized using SEM. Figure 3 shows the SEM images of (a) Fe_3_O_4_ NPs, (b) ZnO NPs, (c) 2,3-Nc, (d) 29H,31H-Pc, and (e) MWCNT; while Fig. 4 shows the SEM images of (a) MWCNT/ZnO/2,3-Nc, (b) MWCNT/Fe_3_O_4_/2,3-Nc, (c) MWCNT/ZnO/29H,31H-Pc and (d) MWCNT/Fe_3_O_4_/29H,31H-Pc. SEM pictures showed that the Fe_3_O_4_ (a) and ZnO (b) particles appeared uniformly distributed forming clustered spherical structures and aggregated particles/rods especially for the ZnO. The average particle size for Fe_3_O_4_ range from 6–25 nm while that of ZnO range from 13–21 nm. The particle size was estimated after calibrating the scale of the SEM image using the UTHSCSA Image Tool for windows version 3.0. 2,3-Nc (c) and 29H,31H-Pc (d) pictures showed an irregularly shaped, small rod-like structures, or clustered flakes, none-uniform in both size and shape. The SEM picture of (e) showed aggregation tubes of the MWCNTs.

The SEM images of MWCNT/ZnO/2,3-Nc (a), MWCNT/Fe_3_O_4_/2,3-Nc (b), showed a tangled-net like structure of MWCNTs embedding the metal oxides in the pores and around its wall. The ZnO nanorods were almost homogeneously distributed in the Pc and the MWCNT forming a more conducting film. This may probably be due to robust ionic and intermolecular interaction between the MO nanoparticles, π-π electrons of the MWCNT and the π-π electrons or lone pair of electron on the nitrogen atoms of the Pc molecule^50^. On the other hand, the SEM pictures of MWCNT/ZnO/29H,31H-Pc (c) and MWCNT/Fe_3_O_4_/29H,31H-Pc (d) composite indicated that the MO nanoparticles formed an aggregate around the MWCNT probably due to less intermolecular π-π interactions between MO NPs, Pc/Nc and MWCNT. It should be noted that 29H,31H-Pc has less π-π electron system compare with Nc. More evidence of their clear distribution on the GCE is shown in supplementary TEM image Figure (S1).

### Electrocatalytic oxidation of epinephrine (EP) and norepinephrine (NE)

**Figure 5:**
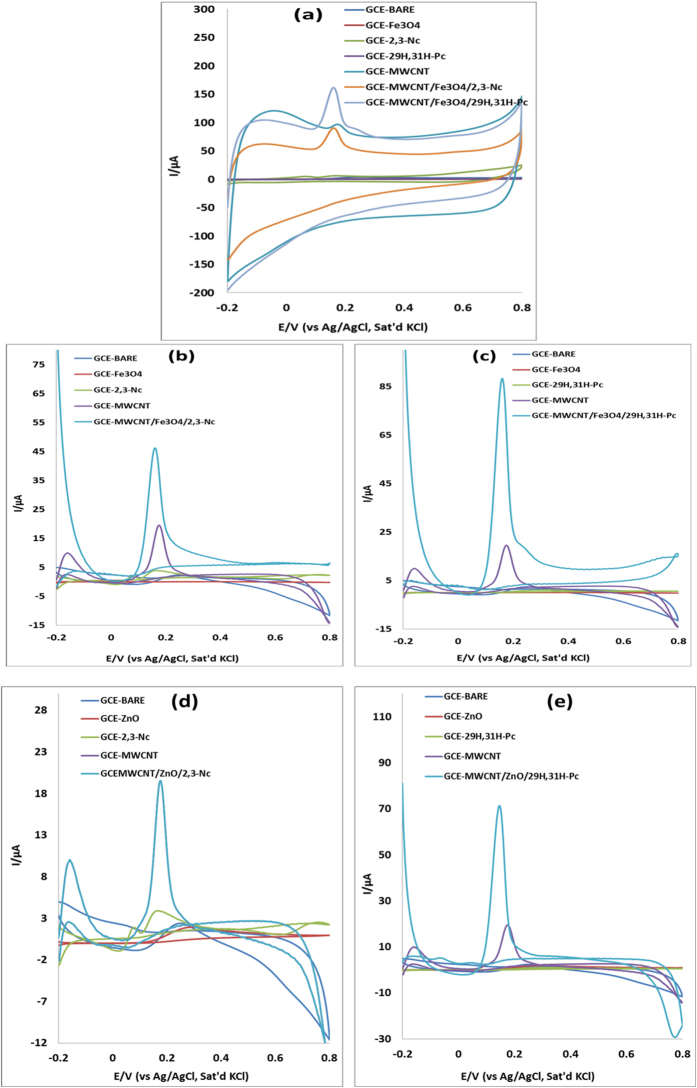
Cyclic voltammograms of bare and modified electrodes in pH 7.2 PBS containing 0.14 mM of EP at scan rate of 25 mVs^−1^ (**a**) (before background current subtraction); (**b–e**) (after background current subtraction).

**Figure 6:**
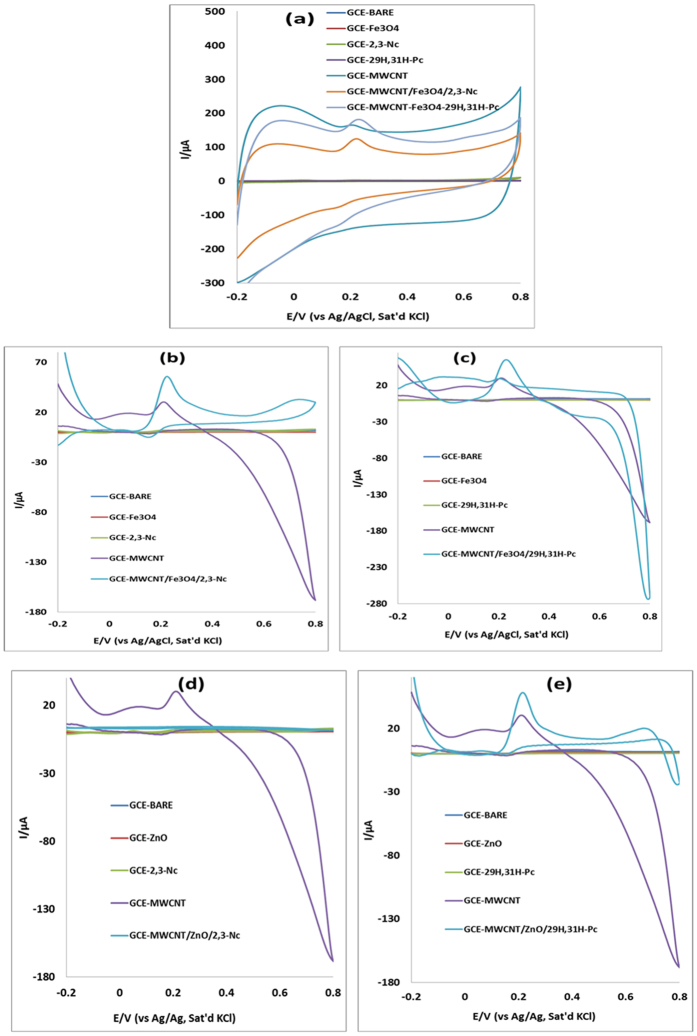
Cyclic voltammograms of bare and modified electrodes in pH 7.2 PBS containing 0.12 mM of NE at scan rate of 25 mVs^−1^ (**a**) (before background current subtraction); (**b–e**) (after background current subtraction).

The electrocatalytic behavior of EP and NE at the bare and modified electrodes was investigated in pH 7.2 PBS containing 0.14 mM of EP and 0.12 mM NE using CV as shown in Figs 5 and 6. Although broad EP and NP peaks were observed at these electrodes, due to the obstruction of flow of electrons, they gave low current response as compared to the bare electrode. No oxidation peak was observed for EP and NE at GCE-Fe_3_O_4,_ probably due to the passive oxide layers which are obstructive to the flow of electrons. It is well known that MWCNT have high electrical conductivity and large surface area, hence electrodes modified with MWCNT conveyed an enhanced current response, which might be due to the possibility of π-π interaction between the aromatic ring of EP and NE and the MWCNT^51^.

However electrodes modified with MWCNT/Fe_3_O_4_/2,3-Nc, MWCNT/Fe_3_O_4_/29H,31H-Pc, MWCNT/ZnO/2,3-Nc and MWCNT/ZnO/29H,31H-Pc showed higher EP oxidation current. The EP peak current at MWCNT/Fe_3_O_4_/29H,31H-Pc electrode was 36 times higher than of the bare electrode and that at MWCNT/ZnO/29H,31H-Pc was 29 times higher. Similarly, MWCNT/ZnO/2,3-Nc and MWCNT/Fe_3_O_4_/2,3-Nc gave NE current response that are 24 and 23 times higher than that at the bare electrode. The large oxidation currents and low oxidation potentials exhibited by these electrodes suggest that the nanocomposites have enhanced electrocatalytic behaviour towards oxidation of EP and NE respectively compared to the bare GCE. The increase in oxidation peak current values can be correlated with the enhanced adsorption capability of EP in the phthalocyanine layer^52^. Furthermore it has been reported that a sharper and well-defined peak and a considerable current increase is due to fast electron transfer^53^. This result agreed with other reports where the enhancement of the chemical sensors was as a result of the carbon nanotubes acting as an electrical conducting nanowire between the modifier and the base electrode^54^,^55^,^56^,^57^. All fabricated electrodes showed an electrochemically irreversible process towards detection of EP and NE. Further studies in this work were carried out using MWCNT/Fe_3_O_4_/2,3-Nc, MWCNT/Fe_3_O_4_/29H,31H-Pc, MWCNT/ZnO/2,3-Nc and MWCNT/ZnO/29H,31H-Pc electrodes

### Stability studies

**Figure 7:**
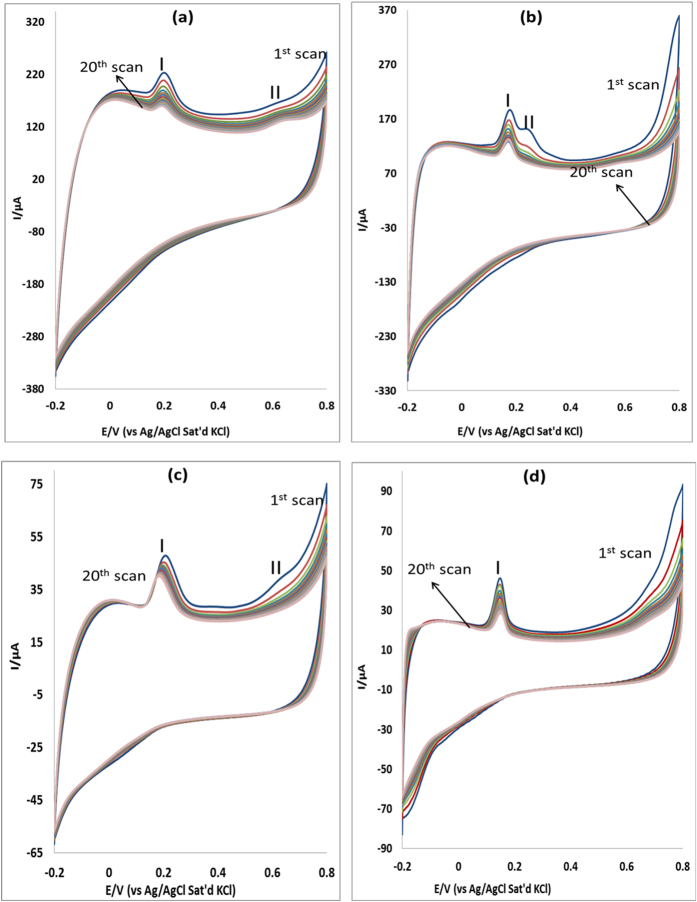
Current response (20 scans) of (**a**) MWCNT/Fe_3_O_4_/2,3-Nc, (**b**) MWCNT/Fe_3_O_4_/29H,31H-Pc, (**c**) MWCNT/ZnO/2,3-Nc and (**d**) MWCNT/ZnO/29H,31H-Pc in pH 7.2 PBS containing 0.14 mM of EP at scan rate of 25 mVs^−1^.

**Figure 8:**
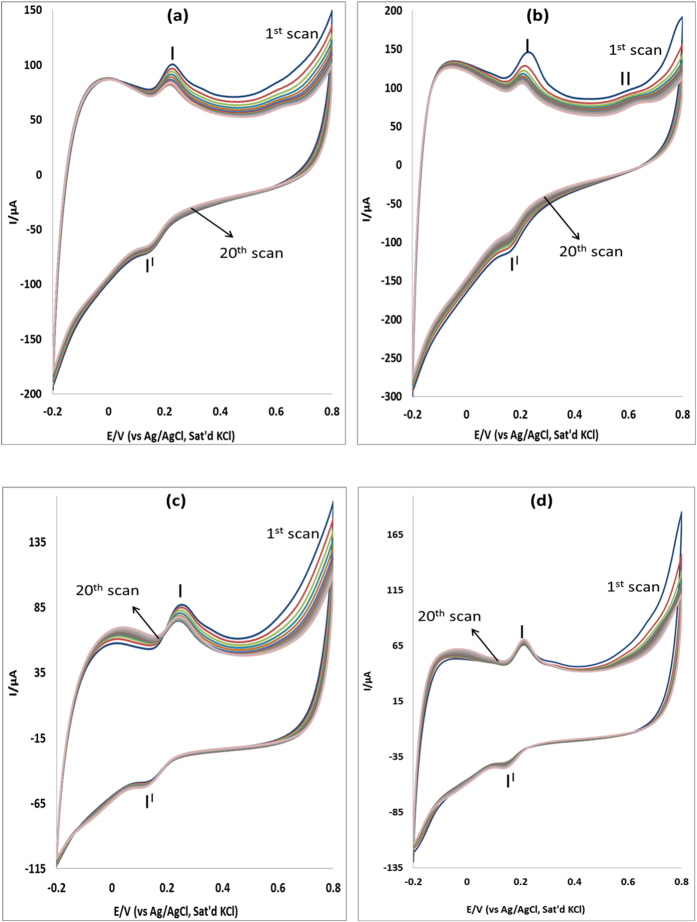
Current response (20 scans) of MWCNT/Fe_3_O_4_/2,3-Nc, MWCNT/Fe_3_O_4_/29H,31H-Pc, MWCNT/ZnO/2,3-Nc and MWCNT/ZnO/29H,31H-Pc in pH 7.2 PBS containing 0.12 mM of NE at scan rate of 25 mVs^−1^.

The stability of MWCNT/Fe_3_O_4_/2,3-Nc, MWCNT/Fe_3_O_4_/29H,31H-Pc, MWCNT/ZnO/2,3-Nc and MWCNT/ZnO/29H,31H-Pc modified electrodes towards determination of EP and NE was studied at 20 successive scans using CV in pH 7.2 PBS containing 0.14 mM of EP and 0.12 mM NE at scan rate of 25 mVs^−1^ (Figs 7 and 8). It was found that the peak current of the first cycle was more than the peak current of the second cycle and after the third cycle there was a minimal decrease in current response. This behavior was observed with all the four modified electrodes. The percentage EP current drop obtained was 5.14%, 5.55%, 5.55% and 5.55% for MWCNT/Fe_3_O_4_/2,3-Nc, MWCNT/Fe_3_O_4_/29H,31H-Pc, MWCNT/ZnO/2,3-Nc and MWCNT/ZnO/29H,31H-Pc modified electrodes respectively. Similarly, the percentage NE current drop are 5.56%, 5.88%, 5.56% and 5.56% for MWCNT/Fe_3_O_4_/2,3-Nc, MWCNT/Fe_3_O_4_/29H,31H-Pc, MWCNT/ZnO/2,3-Nc and MWCNT/ZnO/29H,31H-Pc modified electrodes respectively. Thus the anodic peak currents remained almost stable, indicating excellent electrodes stability during repeated CV scans. These results suggest that there is some level of adsorption of EP and NP at electrodes surface^31^. The adsorptive nature of electrodes might be due to the porous structured layer of the CNTs on the electrodes surface, The RSD obtained for MWCNT/Fe_3_O_4_/2,3-Nc, MWCNT/Fe_3_O_4_/29H,31H-Pc, MWCNT/ZnO/2,3-Nc and MWCNT/ZnO/29H,31H-Pc modified electrodes was 15%, 16%, 2.04% and 5.19% respectively, indicating that these electrodes are relatively stable and are not subjected to serious surface fouling. The oxidation peaks observed in Fig. 7(a,b) might be attributed to oxidation of Fe(II) of Fe_3_O_4_ to Fe(III) and the oxidation peak in Fig. 7(c) might be attributed to the oxidation of ZnO to Zn(OH)_2_. GCE-MWCNT/ZnO/2,3-Nc showed the lowest RSD value as compared to other electrodes, indicating that it was the most stable electrode in determination of EP. Similarly MWCNT/ZnO/29H,31H-Pc modified electrodes is most stable towards NE oxidation having the lowest RSD values of 2.05%.

### Scan rate effect

**Figure 9:**
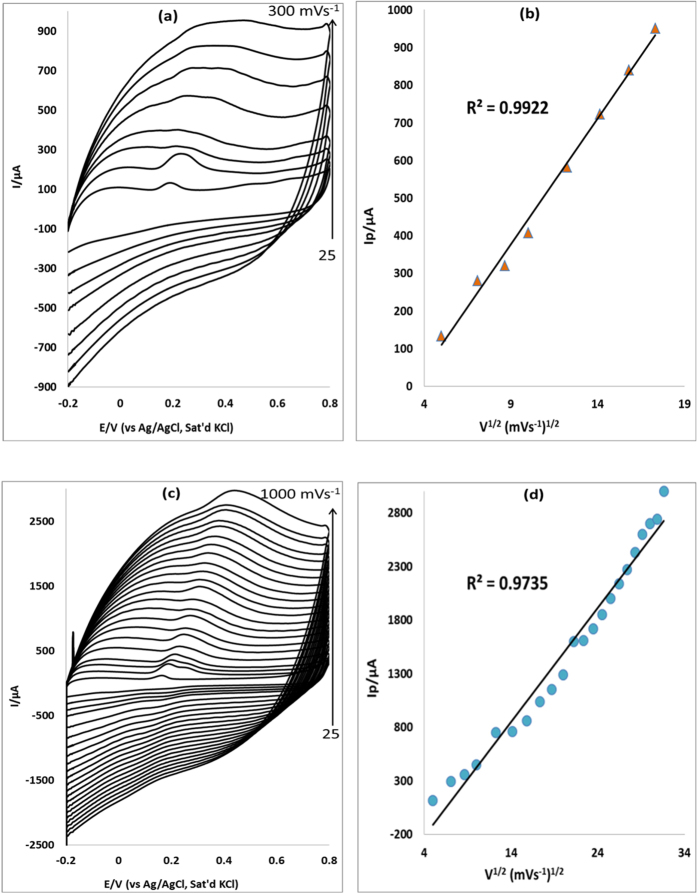
Cyclic voltammograms of (**a**) MWCNT/Fe_3_O_4_/2,3-Nc and (**c**) MWCNT/Fe_3_O_4_/29H,31H-Pc modified electrodes at scan rate (range 25–300 mVs^−1^) and (range 25–1000 mVs^−1^) respectively in pH 7.2 PBS containing 0.14 mM of EP. (**b,d**) are peak current vs. square root of scan rate plots of MWCNT/Fe_3_O_4_/2,3-Nc and MWCNT/Fe_3_O_4_/29H,31H-Pc electrodes respectively.

**Figure 10:**
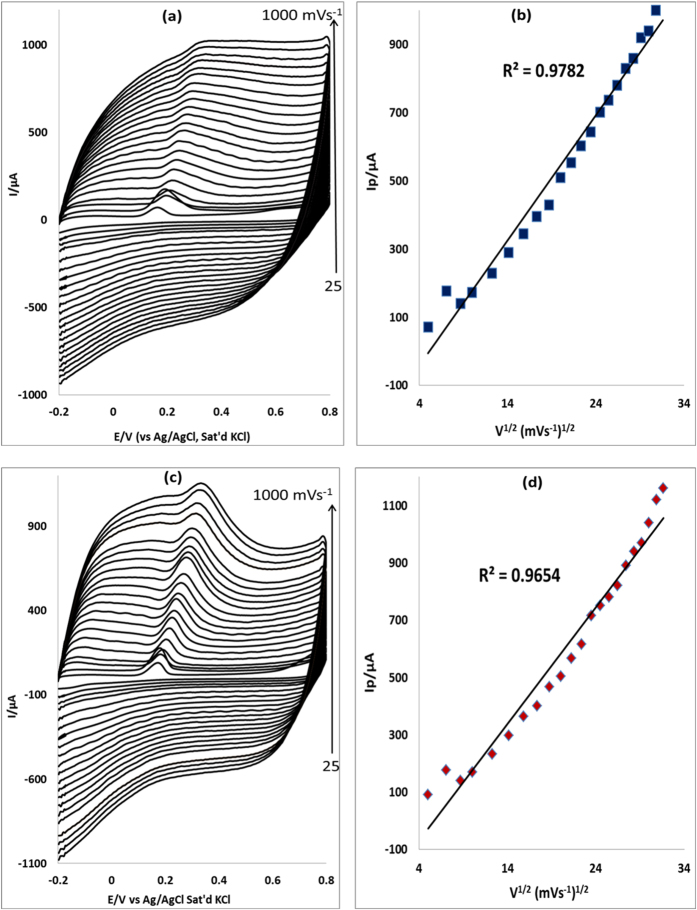
Cyclic voltammograms of (**a**) MWCNT/ZnO/2,3-Nc and (**c**) MWCNT/ZnO/29H,31H-Pc modified electrodes at scan rate range 25–1000 mVs^−1^ in pH 7.2 PBS containing 0.14 mM of EP. (**b,d**) are peak current vs. square root of scan rate plots of MWCNT/ZnO/2,3-Nc and MWCNT/ZnO/29H,31H-Pc respectively.

The effect of scan rate on the anodic peak current of EP and NE was studied at the surface of electrodes modified with MWCNT/Fe_3_O_4_/2,3-Nc, MWCNT/Fe_3_O_4_2/9H,31H-Pc, MWCNT/ZnO/2,3-Nc and MWCNT/ZnO/29H,31H-Pc at a constant concentration of EP (0.14 mM) (Figs 9a,c and 10a,c) and NE (0.12 mM) (not shown) using cyclic voltammetry. Figure 9(b,d) are peak current vs. square root of scan rate plots of MWCNT/Fe_3_O_4_/2,3-Nc and MWCNT/Fe_3_O_4_/29H,31H-Pc electrodes respectively. Similarly, Fig. 10(b,d) are peak current vs. square root of scan rate plots of MWCNT/ZnO/2,3-Nc and MWCNT/ZnO/29H,31H-Pc electrodes respectively. The plots of oxidation peak current vs. scan rate (ν) showed a linear relationship, indicating that the oxidation peak current increased linearly with the increasing scan rate. GCE- MWCNT/Fe_3_O_4_/2,3-Nc (a) showed the EP peak current up to 300 mVs^−1^ although the scan rate was studied at the range of 25–1000 mVs^−1^. Similar results were obtained in NE (not shown). The electrode could not further detect EP and NE over increasing scan rate, indicating saturation of the EP and NE at the electrode surface caused by adsorption of the porous catalyst film^58^. No cathodic peak currents were observed, meaning reaction processes were irreversible. The electrodes gave a negative intercepts from the current versus square root of scan rate plot, indicating an adsorption process^58^. From the plot of peak current I_p_ versus the square root of scan rate, the surface coverage of EP was calculated using Laviron equation (Eq. 1).


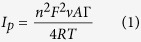


where *n* is the number of electron transfer, *R*, *T* and *F* are the molar gas constant, absolute temperature, and Faraday constant, respectively, *ν* is the scan rate and *Γ* is the surface coverage. Assuming that n ≈ 2 and other parameters have their usual meaning^59^, the surface coverage values of EP were found to be 2 × 10^−8^ mol cm^−2^, 1.76 × 10^−8^ mol cm^−2^, 1.07 × 10^−8^ mol cm^−2^ and 1.37 × 10^−8^ mol cm^−2^ for electrodes modified with MWCNT/Fe_3_O_4_/2,3-Nc, MWCNT/Fe_3_O_4_/29H,31H-Pc, MWCNT/ZnO/2,3-Nc and MWCNT/ZnO/29H,31H-Pc composite respectively. Similarly the surface coverage values obtained for NE are 1.823 × 10^−8^ mol cm^−2^, 1.87 × 10^−8^ mol cm^−2^, 1.432 × 10^−8^ mol cm^−2^ and 1.386 × 10^−7^ mol cm^−2^ for MWCNT/Fe_3_O_4_/2,3-Nc, MWCNT/Fe_3_O_4_/29H,_3_1H-Pc, MWCNT/ZnO/2,3-Nc and MWCNT/ZnO/29H,31H-Pc electrodes respectively.

**Figure 11:**
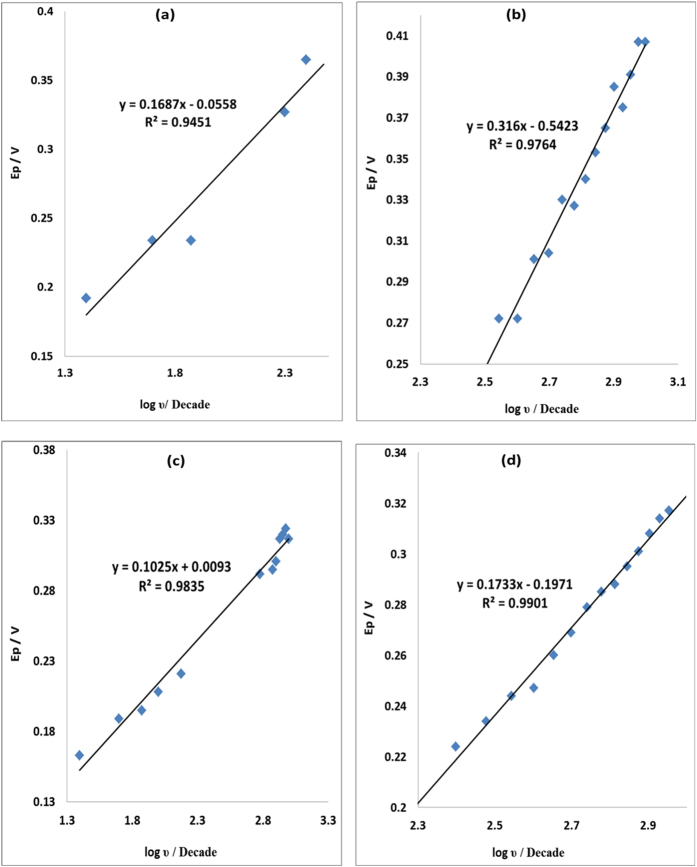
Plots of peak potential (E_p_) versus log υ of (**a**) MWCNT/Fe_3_O_4_/2,3-Nc, (**b**) MWCNT/Fe_3_O_4_/29H,31H-Pc (**c**) MWCNT/ZnO/2,3-Nc and (**d**) MWCNT/ZnO/29H,31H-Pc in pH 7.2 PBS containing 0.14 mM of EP.

The Tafel values were calculated using equation 2 where E_p_ was plotted against log ν (Fig. 11). The obtained values for MWCNT/Fe_3_O_4_/2,3-Nc, MWCNT/Fe_3_O_4_/29H,31H-Pc, MWCNT/ZnO/2,3-Nc and MWCNT/ZnO/29H,31H-Pc modified electrodes in EP were 337.4, 632, 205 and 346.4 mVdec^−1^ respectively. The values are 207.8, 931.8, 1016.4 and 396.6 mVdec^−1^ for MWCNT/Fe_3_O_4_/2,3-Nc, MWCNT/Fe_3_O_4_/29H,31H-Pc, MWCNT/ZnO/2,3-Nc and MWCNT/ZnO/29H,31H-Pc modified electrodes respectively in NE. The high Tafel values were due to the adsorption of the analyte on the electrode surface caused by electrode porosity^60^. The shift in peak potentials as the scan rate increases suggested that EP was oxidized by means of irreversible adsorption process^61^.


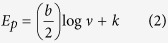


where Ep is the peak potential (V), ν is the scan rate and b represents the Tafel value.

### Concentration study

**Figure 12:**
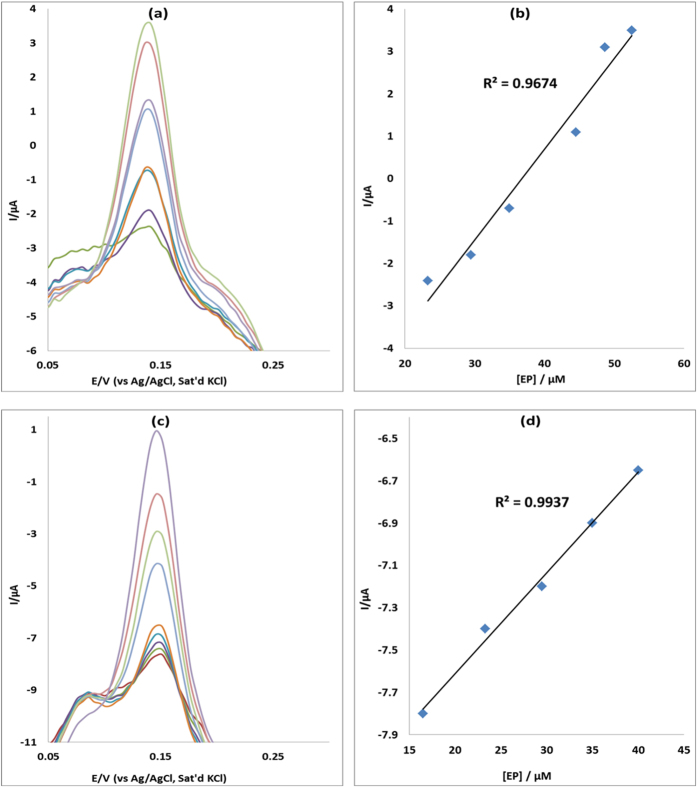
Differential pulse voltammograms of (**a**) MWCNT/Fe_3_O_4_/2,3-Nc and (**c**) MWCNT/Fe_3_O_4_/29H,31H-Pc modified electrodes at scan rate range 25 mVs^−1^ in pH 7.2 PBS containing different concentrations of EP (7.5, 16.5, 23.3, 29.5, 35, 40, 44.5, 48.7, 52.5 and 56 μM; inner to outer). (**b,d**) are peak current vs. concentration of EP plots using MWCNT/Fe_3_O_4_/2,3-Nc and MWCNT/Fe_3_O_4_/29H,31H-Pc electrodes respectively.

**Figure 13:**
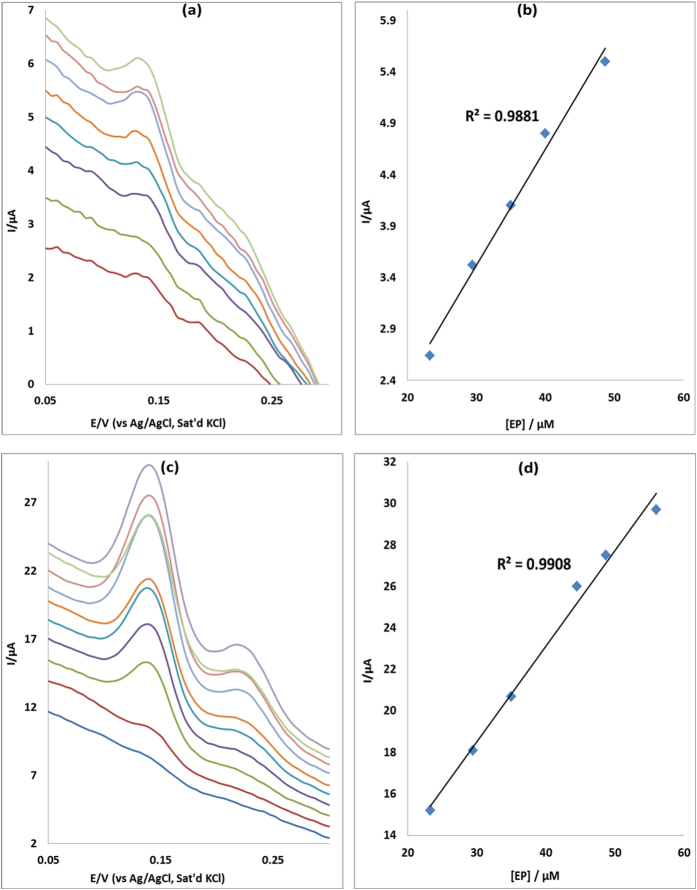
Differential pulse voltammograms of (**a**) MWCNT/ZnO/2,3-Nc and (**c**) MWCNT/ZnO/29H,31H-Pc modified electrodes at scan rate range 25 mVs^−1^ in pH 7.2 PBS containing different concentrations of EP (7.5, 16.5, 23.3, 29.5, 35, 40, 44.5, 48.7, 52.5 and 56 μM; inner to outer). (**b,d**) are peak current vs. EP concentration plots using MWCNT/ZnO/2,3-Nc and MWCNT/ZnO/29H,31H-Pc electrodes respectively.

**Figure 14:**
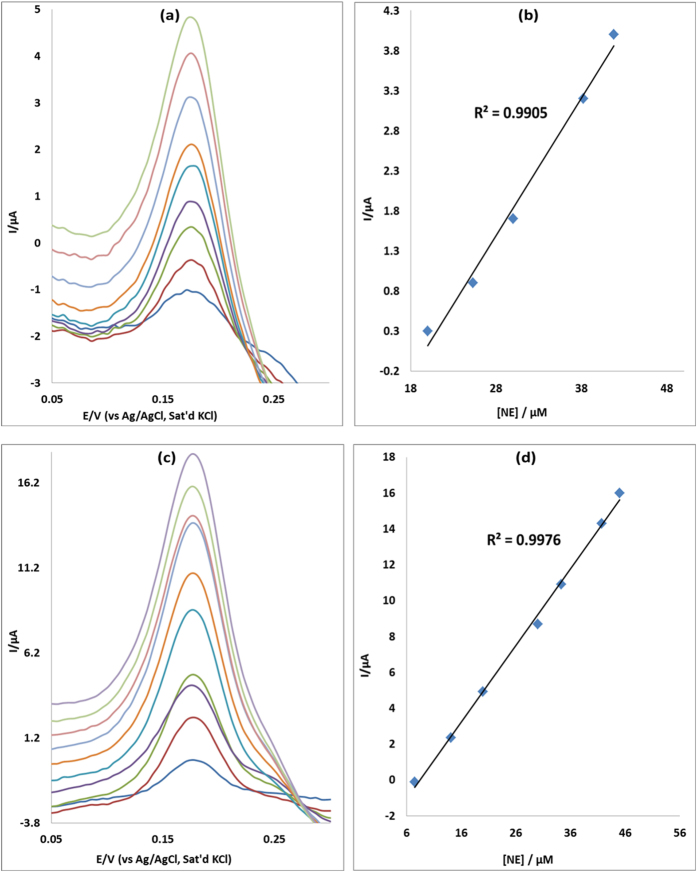
Differential pulse voltammograms of (**a**) MWCNT/Fe_3_O_4_/2,3-Nc and (**c**) MWCNT/Fe_3_O_4_/29H,31H-Pc modified electrodes at scan rate range 25 mVs^−1^ in pH 7.2 PBS containing different concentrations of NE (7.5, 14.1, 20, 25.3, 30, 34.3, 38.2, 41.7, 45 and 48 μM; inner to outer). (**b,d**) are peak current vs. NE concentration plots using MWCNT/Fe_3_O_4_/2,3-Nc and MWCNT/Fe_3_O_4_/29H,31H-Pc electrodes respectively.

**Figure 15:**
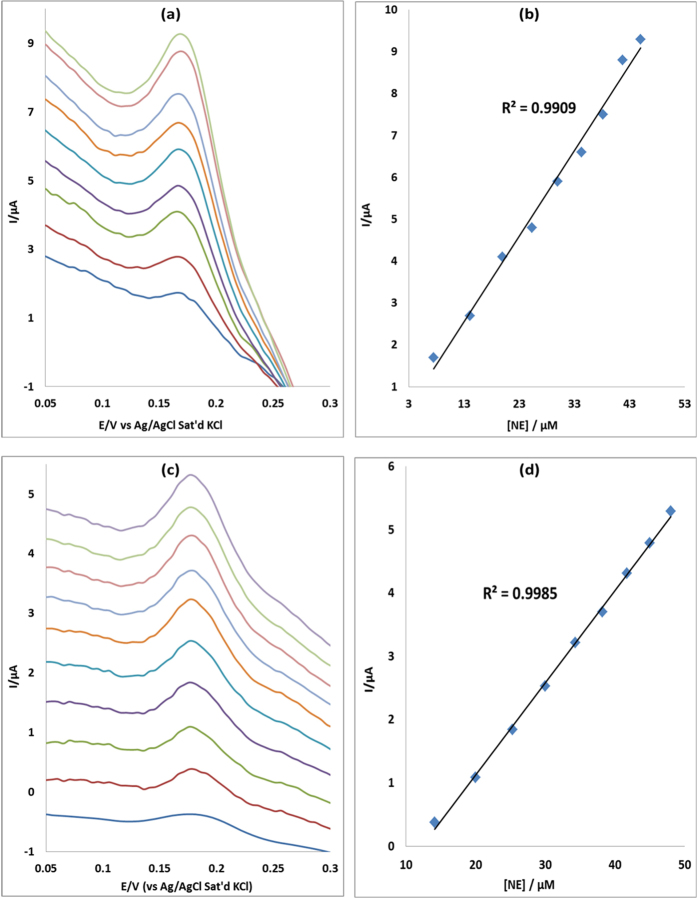
Differential pulse voltammograms of (**a**) MWCNT/ZnO/2,3-Nc and (**c**) MWCNT/ZnO/29H,31H-Pc modified electrodes at scan rate range 25 mVs^−1^ in pH 7.2 PBS containing different concentrations of NE (7.5, 14.1, 20, 25.3, 30, 34.3, 38.2, 41.7, 45 and 48 μM; inner to outer). (**b,d**) are peak current vs. NE concentration plots using MWCNT/ZnO/2,3-Nc and MWCNT/ZnO/29H,31H-Pc electrodes respectively.

The effect of concentration of EP was investigated using differential pulse voltammetry (DPV) on MWCNT/Fe_3_O_4_/2,3-Nc, MWCNT/Fe_3_O_4_/29H,31H-Pc, MWCNT/ZnO/2,3-Nc and MWCNT/ZnO/29H,31H-Pc modified electrodes. The study was carried for EP concentration range of 7.5–56 μM in pH 7.2 PBS. Figures 12 and 13 show the linear variation of peak currents with concentration of EP and calibration plots of current versus concentration obtained. Similar plots were obtained for NE (Figs 14 and 15). The results obtained showed that the oxidation current peaks are directly proportional to increase in EP concentration. It was found to show satisfactory linearity over a range of concentrations from 7.5–48 μM. The detection limit was calculated based on the relationship LoD = 3.3 δ/m. Where *δ* is the relative standard deviation of the intercept of the y-coordinates from the line of best fit, and *m* the slope of the same line^62^. The detection limits of 12.3, 4.6, 7.6 and 6.5 μM were obtained for EP at MWCNT/Fe_3_O_4_/2,3-Nc, MWCNT/Fe_3_O_4/_29H,31H-Pc, MWCNT/ZnO/2,3-Nc and MWCNT/ZnO/29H,31H-Pc modified electrodes respectively. Similarly, NE detection limit of 6, 2.2, 3.6 and 1.7 μM was obtained for MWCNT/Fe_3_O_4_/2,3-Nc, MWCNT/Fe_3_O_4_/29H,31H-Pc, MWCNT/ZnO/2,3-Nc and MWCNT/ZnO/29H,31H-Pc modified electrodes respectively. From these results it can be seen that MWCNT/Fe_3_O_4_/29H,31H-Pc modified electrode gave the lowest limit of detection with low sensitivity of 0.048 μA/μM to EP as compared to the high sensitivity of MWCNT/Fe_3_O_4_/2,3-Nc (0.214 μA/μM) and MWCNT/ZnO/29H,31H-Pc (0.461 μA/μM) modified electrodes. Mazloum-Ardakani *et al*.^63^ mentioned that the decrease in sensitivity might be due to kinetic limitations. In addition it has been mentioned that high sensitivity won’t necessarily equate to a low limit of detection, but a decrease in matrix interference (increasing selectivity) and high background noise. MWCNT/ZnO/29H,31H-Pc modified electrode also gave the lowest limit of detection towards NE. The limit of detection obtained in this work compared with others reported in literature^57^,^64^. Incidentally, both the GCE-MWCNT/Fe_3_O_4_/29H,31H-Pc and GCE-MWCNT/ZnO/29H,31H-Pc modified electrodes that gave the lowest limit of detection also gave the best catalysis in terms of current response and onset potential of catalysis towards EP and NE respectively.

### Interference study

**Figure 16:**
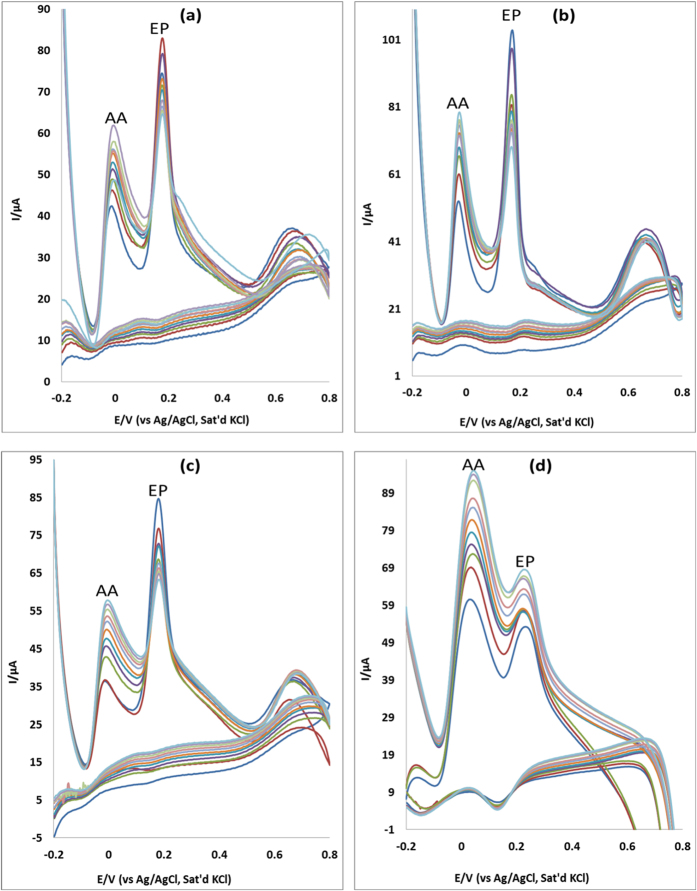
Cyclic Voltammograms of binary mixture of EP and AA at (**a**) MWCNT/Fe_3_O_4_/2,3-Nc, (**b**) MWCNT/Fe_3_O_4_/29H,31H-Pc, (**c**) MWCNT/ZnO/2,3-Nc and (**d**) MWCNT/ZnO/29H,31H-Pc with constant concentration of EP (0.14 mM) and increasing concentration of AA (0.32, 0.58, 0.81, 1.0, 1.2, 1.3, 1.4, 1.6, 1.7, 1.8 mM).

**Figure 17:**
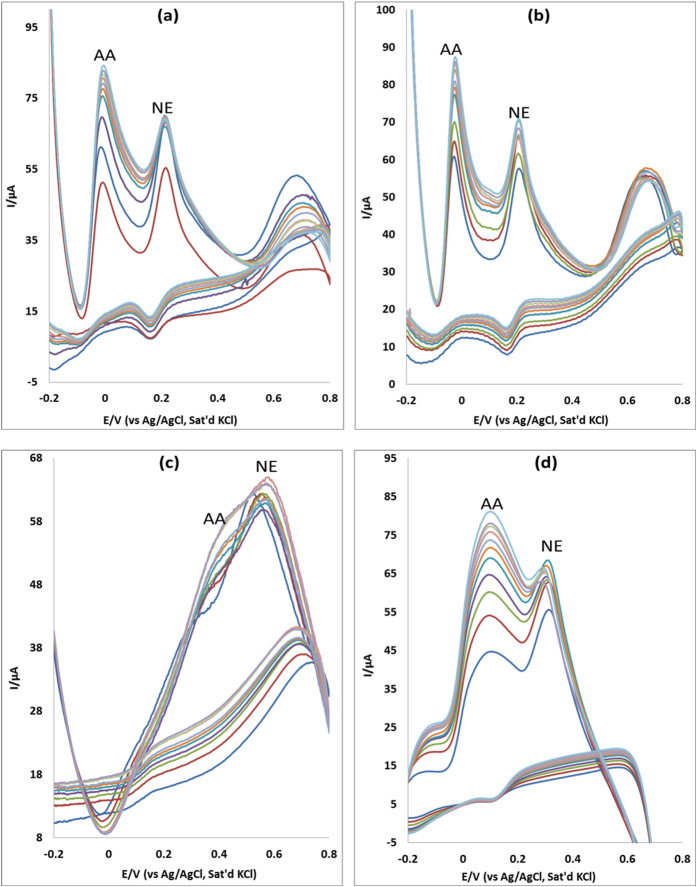
Cyclic voltammograms of binary mixture of NE and AA at (**a**) MWCNT/Fe_3_O_4_/2,3-Nc, (**b**) MWCNT/Fe_3_O_4_/29H,31H-Pc, (**c**) MWCNT/ZnO/2,3-Nc and (**d**) MWCNT/ZnO/29H,31H-Pc with constant concentration of NE (0.12 mM) and increasing concentration of AA (0.32, 0.58, 0.81, 1.0, 1.2, 1.3, 1.4, 1.6, 1.7, 1.8 mM).

AA is an electroactive species known to coexist and get oxidized at similar potentials with EP in biological systems at most modified electrodes. Hence it is significant to selectively detect EP in the presence of AA because these analytes show an overlap of signals at some chemically modified electrodes. The selectivity and sensitivity of MWCNT/Fe_3_O_4_/2,3-Nc, MWCNT/Fe_3_O_4_/29H,31H-Pc, MWCNT/ZnO/2,3-Nc and MWCNT/ZnO/29H,31H-Pc modified electrodes was investigated using cyclic voltammetry, with increasing concentration of AA (0.32, 0.58, 0.81, 1.0, 1.2, 1.3, 1.4, 1.6, 1.7, 1.8 mM) while the concentration of EP (0.14 mM) and NE (0.12 mM) was kept constant. In Fig. 16, the four electrodes showed two well distinguished oxidations peaks of AA and EP and at AA concentration which is 30-fold excess of EP, no significant interference of EP signal due to AA was observed. Similar results were obtained for NE (Fig. 17). The difference between peak potentials of EP and NE from AA is summarized in Tables 1 and 2. The anodic peak signals of AA and EP were independent from each other; therefore the modified electrodes were able to adequately identify the two analytes. The simultaneous determination of AA and EP using the four modified electrodes was achieved, indicating that the developed sensors can be suitably used to determine EP in biological fluids. However MWCNT/Fe_3_O_4_/2,3-Nc, MWCNT/ZnO/2,3-Nc and MWCNT/ZnO/29H,31H-Pc modified electrodes showed big potential peak separation as compared to MWCNT/Fe_3_O_4_/29H,31H-Pc modified electrode. The simultaneous determination of AA and NE was achieved with only three electrodes, suggesting that those electrodes can be suitably used to determine NE in biological fluids.

## Conclusion

This study investigated the electrocatalytic behaviour of GCE modified with MWCNT, ZnO, Fe_2_O_3_, Pc, Nc, MWCNT/Fe_3_O_4_/2,3-Nc, MWCNT/Fe_3_O_4_/29H,31H-Pc, MWCNT/ZnO/2,3-Nc and MWCNT/ZnO/29H,31H-Pc towards epinephrine (EP) and norepinephrine (NE) oxidation. The catalysis of EP and NP was more favoured on the GCE-MWCNT/Fe_3_O_4_/2,3-Nc, GCE-MWCNT/Fe_3_O_4_29H,31H-Pc, GCE-MWCNT/ZnO/2,3-Nc and GCE-MWCNT/ZnO/29H,31H-Pc electrodes with enhanced epinephrine and norepinephrine current response compared to other electrodes. The enhanced current response of these electrodes was attributed to the synergy between the MWCNTs, MO NPs and the phthalocyanines. All the four electrodes showed a good stability with low current drop (5–10%). The catalysis of EP was more favoured on GCE-MWCNT/Fe_3_O_4_/29H,31H-Pc electrode while that of NE was more favored on MWCNT/ZnO/29H,31H-Pc electrode in terms of current response and onset potential for catalysis compared to other electrodes investigated. These two electrodes also gave the lowest limit of detection towards EP and NE respectively. Aside the fact that the modified electrodes gave comparable limit of detection in the micro molar range with literature, they have also demonstrated excellent resistant to electrode fouling effect as demonstrated by very low (5%) EP and NE current drop after long scans and effective separation of the analyte signals from that of AA at potential as large as 200 mV. The electrodes could conveniently detect EP and NE in the presence of AA with a wide potential separation of about 200 mV. The electrodes were found to be electrochemically stable, re-usable and can thus be used for the analysis of EP and NE in real biological samples.

## Additional Information

**How to cite this article**: Mphuthi, N. G. *et al*. Electrocatalytic oxidation of Epinephrine and Norepinephrine at metal oxide doped phthalocyanine/MWCNT composite sensor. *Sci. Rep*. **6**, 26938; doi: 10.1038/srep26938 (2016).

## Supplementary Material

Supplementary Information

## Figures and Tables

**Table 1:** Cyclic voltammetric parameters of binary mixture of EP and AA obtained for modified electrodes with constant concentration of EP (0.14 mM) and increasing concentration of AA.

Fabricated GC electrodes	Anodic peak potentials (mV)		Peak potential separation (V)
	AA	EP	
MWCNT/Fe_3_O_4_/2,3-Nc	−0.006	0.176	0.17
MWCNT/Fe_3_O_4_/29H,31H-Pc	−0.026	0.167	0.141
MWCNT/ZnO/2,3-Nc	−0.006	0.180	0.174
MWCNT/ZnO/29H,31H-Pc	0.042	0.224	0.182

**Table 2:** Cyclic voltammetric of binary mixture of NE and AA obtained for modified electrodes with constant concentration of NE (0.12 mM) and increasing concentration of AA.

Fabricated GC electrodes	Anodic peak potentials (mV)		Peak potential separation (V)
	AA	NE	
MWCNT/Fe_3_O_4_/2,3-Nc	−0.01	0.215	0.205
MWCNT/Fe_3_O_4_/29H,31H-Pc	−0.026	0.208	0.182
MWCNT/ZnO/2,3-Nc	–	–	–
MWCNT/ZnO/29H,31H-Pc	0.078	0.307	0.229
