# Supplementary material for: Electrocatalytic oxidation of Epinephrine and Norepinephrine at metal oxide doped phthalocyanine/MWCNT composite sensor
Source: Sci Rep. 2016 Jun 1;6:26938. doi: 10.1038/srep26938 (PMC4887908; doi:10.1038/srep26938)
Supplement: Supplementary Information [file srep26938-s1.docx]

**Electrocatalytic oxidation of Epinephrine and Norepinephrine at metal oxide doped phthalocyanine/MWCNT composite sensor**

Ntsoaki G. Mphuthi^1,^, Abolanle S. Adekunle^1,2,^, Eno E. Ebenso^1,*^

**
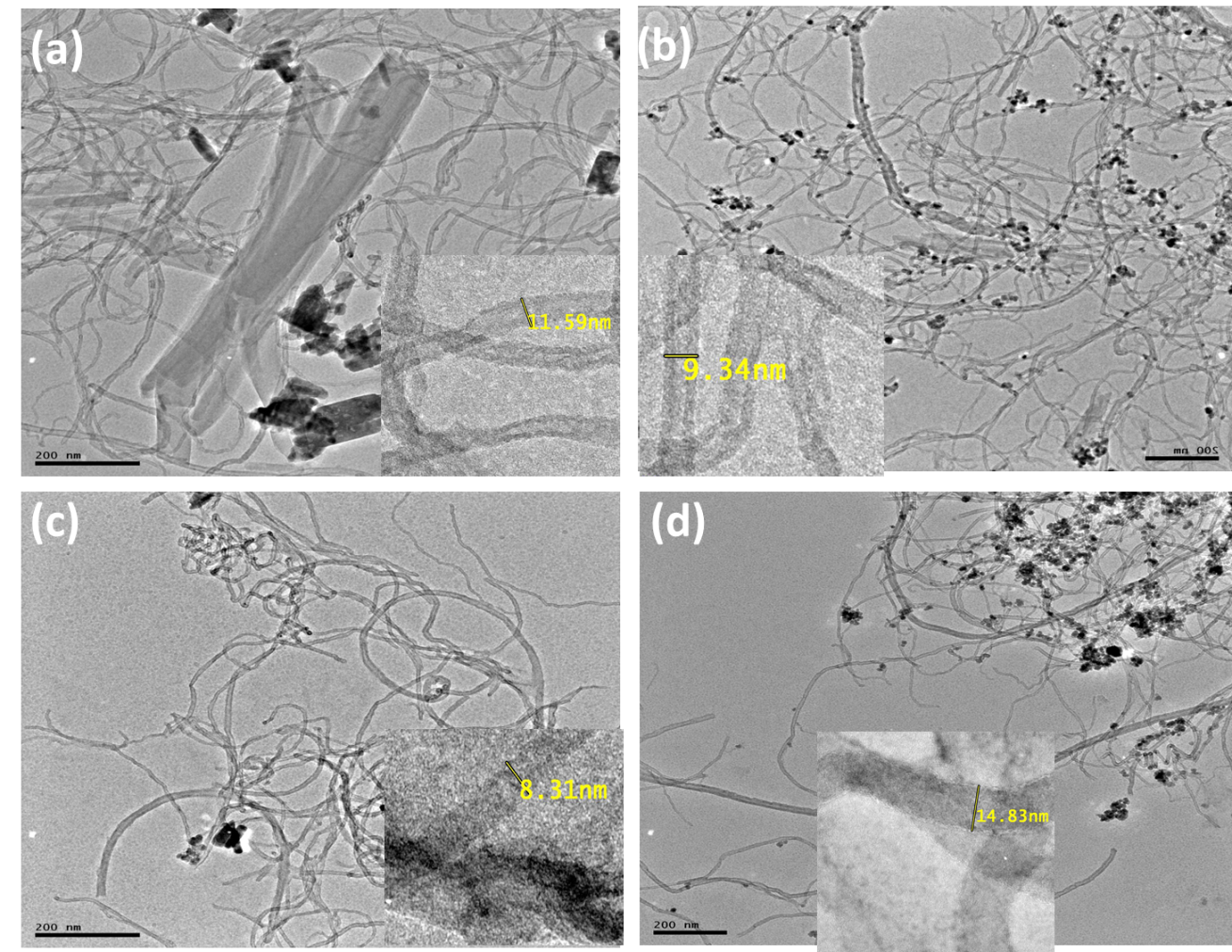
**

**S1:** TEM images of (a) MWCNT/ZnO/2,3-Nc, (b) MWCNT/Fe_3_O_4_/2,3-Nc (c) MWCNT ZnO/29H,31H-Pc and (d) MWCNT/Fe_3_O_4_/29H,31H-Pc.
